# Spectrum Sensing Based on Hybrid Spectrum Handoff in Cognitive Radio Networks

**DOI:** 10.3390/e25091285

**Published:** 2023-08-31

**Authors:** Lakshminarayanan Vaduganathan, Shubhangi Neware, Przemysław Falkowski-Gilski, Parameshachari Bidare Divakarachari

**Affiliations:** 1 Department of Electrical and Electronics Engineering, Dr. Mahalingam College of Engineering and Technology, Pollachi 642003, India; vln@mcet.in; 2 Department of Computer Science and Engineering, Shri Ramdeobaba College of Engineering and Management, Nagpur 440013, India; newares@rknec.edu; 3 Faculty of Electronics, Telecommunications and Informatics, Gdansk University of Technology, Narutowicza 11/12, 80-233 Gdansk, Poland; 4 Department of Electronics and Communication Engineering, Nitte Meenakshi Institute of Technology, Bengaluru 560064, India

**Keywords:** cognitive radio networks, component-specific adaptive estimation, primary users, power spectrum, spectrum sensing

## Abstract

The rapid advancement of wireless communication combined with insufficient spectrum exploitation opens the door for the expansion of novel wireless services. Cognitive radio network (CRN) technology makes it possible to periodically access the open spectrum bands, which in turn improves the effectiveness of CRNs. Spectrum sensing (SS), which allows unauthorized users to locate open spectrum bands, plays a fundamental part in CRNs. A precise approximation of the power spectrum is essential to accomplish this. On the assumption that each SU’s parameter vector contains some globally and partially shared parameters, spectrum sensing is viewed as a parameter estimation issue. Distributed and cooperative spectrum sensing (CSS) is a key component of this concept. This work introduces a new component-specific cooperative spectrum sensing model (CSCSSM) in CRNs considering the amplitude and phase components of the input signal including Component Specific Adaptive Estimation (CSAE) for mean squared deviation (MSD) formulation. The proposed concept ensures minimum information loss compared to the traditional methods that consider error calculation among the direct signal vectors. The experimental results and performance analysis prove the robustness and efficiency of the proposed work over the traditional methods.

## 1. Introduction

The phrase “Spectrum Handoff” or “Spectrum Handover” refers to the procedure used in the cognitive radio (CR) network for users to change spectrum bands. A transceiver can intelligently determine which communication channels are in use and which ones are not in CR, a form of wireless communication [1]. The transceiver then immediately switches to open channels, avoiding busy ones [2]. Moreover, it increases spectrum efficiency and the consumer’s quality of service (QoS) through avoiding occupied channels. With the explosive expansion of wireless communication industries [3], a significant demand exists for establishment of novel wireless networks in licensed and unlicensed frequency spectra. Recent research demonstrates that the current fixed spectral assignment approach leads to subpar spectrum utilization [4,5,6]. Cognitive radio networks (CRNs) have emerged as a viable technique to solve this issue by allowing access to the sporadic intervals of vacant frequency bands, often known as white space or spectrum gaps, and therefore improving spectrum efficiency (SE) [7,8,9]. In the most basic sense, every CR user in a CRN must first determine if licensed users, also known as primary users (PUs), are present and if not, whether the spectrum is accessible. Spectrum sensing (SS) is a kind of radio frequency (RF) environment sensing that is typically used to accomplish this [10,11,12].

SS has two goals: first, CR users must get out of interfering negatively with PUs by moving to an open band to a reasonable level [13,14,15]. Second, to attain the essential throughput and QoS, CR users should effectively locate and utilize the spectrum gaps [16,17,18]. Therefore, the effectiveness of primary and cognitive radio networks depends on the detection accuracy in SS [19,20].

The performance of detection could be determined primarily depending upon two metrics: false alarm (FA) probability indicates the probability of a CR user stating that a PU is available while the spectra are free, and detection probability indicates the probability of CR user portraying that a PU is available while the spectra are indeed engaged by a PU [21]. As a detection miss leads to intervention with PUs and a FA would lessen the SE, it is typically necessary for optimum detection performance where the probability of detection is increasingly subjected to an FA probability [22]. The performance of detection in SS may be considerably hampered by a variety of issues, including receiver uncertainty, shadowing, and multipath fading [23].

The main contributions of this study is as follows.

This study proposed a component-specific cooperative spectrum sensing model (CSCSSM) which considers the amplitude and phase components of the input signal to decrease the information loss in CRNs.

The component-specific adaptive estimation (CSAE) is proposed for calculating the mean squared deviation (MSD).

This paper is structured as follows: Section 2 describes the existing component-specific cooperative spectrum sensing (CSS) models. Section 3 explains the proposed CSAE. The component-specific adaptive estimation (CSAE) for MSD formulation is described in Section 4, whereas Section 5 presents the results. Finally, Section 6 provides the conclusion of this paper.

## 2. Literature Review

### 2.1. Related Works

In 2018, Muthukkumar and Manimegalai [24] examined the collaboration between secondary users (SUs) and main users using the Priority-Based Two-Stage Detection Model (PBTSDM). SUs in distributed CSS continually sensed among themselves and used an entropy-based energy detection approach to jointly determine whether or not PUs were present. The outcomes displayed that applying the suggested technique considerably improved the accuracy of energy efficiency (EE) and sensing time. However, noise uncertainty was a concern.

In 2017, Atmaca et al. [25] used cooperative spectrum sensing to maximize the throughput of Carrier Sense Multiple Access (CSMA) in Random Access CRNs (RACRNs). A CRN was simulated using the CSMA media access control (MAC) system in this study, with a particular emphasis on examining its throughput performance. In the identical network-level condition, throughput performances of CRNs were achieved and compared. Nevertheless, the network load needed to be concentrated more.

In 2019, Sharifi [26] offered an effective protection strategy using the Attack Aware CSS (ACSS). The concept was based on the assessment of attack strength, where attack population and assault strength were correlated. The chance that a particular sensor was malicious is equal to the ratio of malevolent sensors to all sensors, which was known as the attack strength. The suggested method predicted attack strength and used the Bayesian hypothesis test to enhance collaborative sensing performance, supposing malicious sensor activity or an attack plan. However, strong interference might affect PUs.

In 2021, Ye and Jiang [27] proposed a study on cluster-based CRNs that included an ideal linear-scaled CSS. Different weight values for cooperative nodes were assigned in this system depending on the signal-to-noise ratio (SNR) of CR users and the historic sensing accuracy. Additionally, the CR users could be grouped, and the cluster heads chosen to collect the local sensing data were the users with superior channel characteristics. The suggested approach provided superior sensing performance while also increasing detection probability and lowering error probability, according to the simulation findings. More experimental platforms need to be considered to confirm the feasibility of this approach.

In 2021, Devi and Umamaheswari [28] included the use of the M/G/1 queuing model and the Spectrum Binary Particle Swarm Optimization (Spec BPSO) algorithm for the prediction of an efficient spectrum handoff method. Cluster-based CSS (CBCSS) was employed to increase SU effectiveness and decrease channel congestion. This research project also provided a framework for observing how main user behavior affected spectrum handoff performance delays with potential CRN interruptions. Nevertheless, metaheuristic schemes were not focused on.

In 2020, Rajaganapathi and Nathan [29] developed the accurate CSS and optimal relay selection (ORS) system, which enhanced the SUs using a hybrid CRN throughput. The precision of choosing the underlay/overlay technique to convey information was increased by an accurate CSS approach. When an underlying transmission strategy is chosen, SUs employ relays to reduce interference. An optimal relay selection approach was applied in this case to optimize relay choice. The throughput was improved by the suggested system, according to the numerical data. In the future, optimization concepts can be included to ensure more enhanced results.

To effectively use the report time slot by increasing the detecting time of SUs, in 2021, Hossain et al. [30] suggested the idea of Multiple Reporting Channels (MRCs) for clustered CRNs. In this method, each cluster was given a reporting channel for reporting purposes. The designated single reporting channel was used by all the SUs in every cluster to progressively transmit their sensing findings to the associated CH, extending the SUs’ sensing time length. This method considerably improved all SUs’ sensing times compared to non-sequential reporting and also reduced all cluster heads’ (CHs’) reporting time delays compared to sequential single-channel reporting. Multiple PUs as well as ML concepts were not taken into account.

In 2018, Jaglan et al. [31] deployed Artificial Neural Networks (ANNs) at fusion centers, which resulted in a notable improvement in detection accuracy and a decrease in the FA rate when compared to traditional methods. It was determined that the suggested ANN technique can handle CRN scalability while maintaining performance. Additionally, the SNR of each SU was taken into account while making decisions at the fusion center. Furthermore, the suggested method was evaluated for resilience against security attacks (malicious users) and unintentional mistakes happening at SUs. A minimal amount of FA issues occurred.

In 2022, Arshid et al. [32] deployed a user transmission system that senses available channels through cooperative spectrum sensing. Energy economy was achieved by optimizing the energy consumption of the sensing process. For spectrum managing, a threshold method based on main user traffic patterns was presented. A CSS was also explained and executed to find the best channel with the highest throughput and least amount of energy use. The suggested method improved throughput and energy efficiency while maintaining the handoff delay, and preventing false alarms and missed detection.

In 2022, Bani and Kulkarni [33] deployed a hybrid detector (HD) to identify spectrum holes using the available resources. An energy detector (ED) and matched detector (MD) served as the foundation for the HD architecture. The HD was able to sense the signal more accurately than a single detector like an ED. Whether or not the primary user information was accessible in this case, HD functioned under both circumstances. Under heterogeneous conditions, HD was analyzed both with and without spectrum sensing. The IEEE Wireless Regional Area Network (WRAN) 802.22 standard served as the foundation for the HD’s design specifications. OR rules produced the best outcomes for the HD model.

### 2.2. Research Gaps

Users of CR pooled their sensory data through cooperation in order to make judgements that were more accurate when combined than when taken separately. Due to multipath fading and shadowing, the SNR of the received primary signal was very low, making the identification difficult. Since receiver sensitivity is the ability to sense weak signals, the receiver was subjected to strict sensitivity criteria, which greatly increased the implementation complexity and hardware cost.

More crucially, while the SNR of the PU signal was below what is known as an SNR wall, the detecting performance could not be increased by raising the sensitivity. Fortunately, CSS significantly decreased the sensitivity required and the hardware restriction difficulties. CSS was used to alleviate multipath fading- and shadowing-related performance loss without raising the cost of CR device installation. The cooperative advantage, however, extended beyond enhanced detection performance and loosened sensitivity requirements [34].

As was previously said, cooperative sensing led to cooperative gain, but there were a variety of conditions that restricted this benefit. For instance, their observations were coupled when CR users were stopped by the same obstruction and were under spatially correlated shadowing. Cooperation amongst more spatially connected CR users functioned as well for detection. This brought up the question of user selection in cooperative sensing [35].

The influence of nearby SUs’ behavior on an SU was not taken into consideration in the conventional spectrum handoff method; additionally, the spectrum handoff condition in a single field was only carried out in CRNs [36] and the hybrid spectrum access setup merging interweave mode by underlay/m-mode which was not discussed here. Thus, a paradigm is suggested to address the inadequacies of the aforementioned existing spectrum handoff methodologies.

## 3. Component-Specific CSS Model

Spectrum handoff is regarded as the primary problem in spectrum mobility when a PU appears and SUs use this specific PU as a licensed channel. Spectrum handoff is an essential part of CRNs that enables resilient service for secondary consumers and is designed to assist secondary users in locating suitable target channels to carry out communication. The proposed CSCSSM model manages transmission power and chooses the channels with the longest holding time to avoid the spectrum handoff.

Assume P to be PUs and S to be SUs. The power spectrum discharged by every PU is captured as a linear grouping of certain basic operations. Now, Gaussian is used as a base operation. Every SU, through SS, effectively identifies the entire spectrum from every PU region. The power spectrum from PU p is modeled in Equation (1).

(1) $$\begin{array}{l} K_{p}=\sum_{m=1}^{A}a_{pm}g_{m}\left(e^{j\omega }\right)\\ =g_{\omega }\varpi_{p},\;\;p=1,2.....P \end{array}$$

In Equation (1), A refers to the number of CRs present in the network;$\;K_{p}$ refers to the summation of signals received at each CH; $g_{m}\left(e^{j\omega }\right)=e^{-\frac{\left(\omega -\omega_{m}\right)}{2\sigma_{m}^{2}}}$ and constraints $\omega_{m}$, $\sigma_{m}$ refer to the central frequency and standard deviation; $g_{\omega }=\left[g_{1}\left(e^{j\omega }\right),g_{2}\left(e^{j\omega }\right),g_{3}\left(e^{j\omega }\right)\ldots g_{A}\left(e^{j\omega }\right)\;\right]$ refers to a vector with base operations; scalars $\left\{a_{pm}\right\}$ refer to coefficients of the base extension for user p; and $\varpi_{p}=\left[a_{p_{1}},a_{p_{2}}\ldots ,a_{p_{A}}\right]$ refers to a vector with aspects involved in the linear grouping of the base operations. Equation (1) can estimate the necessary part of the power spectra if A is adequate.

The power spectra from SU s is identified through PU p which is attenuated owing to transmission path loss implied by $q_{ps}$. The path loss coefficient is identified and described earlier in a training phase among PUs by every SU. Training is typically repetitive at certain periods since the coefficients vary (gradually) in time, owing to the movement of the node. If the broadcasted spectrum moves from PU to SU, the previous power spectra are evaluated by the receiver of the SU s, denoted as $q_{ps}K_{p}\left(e^{j\omega }\right)$. Therefore, the entire power spectra from every PU at SU s are modeled as in Equation (2).

(2) $$\begin{array}{l} K_{s}^{t}=\sum_{p=1}^{P}q_{ps}K_{p}\left(e^{j\omega }\right)+\sigma_{s}^{2}\\ =\sum_{p=1}^{P}q_{ps}g_{\omega }\varpi_{p}+\sigma_{s}^{2}\\ =v_{s,\omega }\varpi_{s}^{o}+\sigma_{s}^{2} \end{array}$$

In Equation (2), $\varpi_{s}^{o}={\left[\varpi_{1}^{T},\varpi_{2}^{T}\ldots \varpi_{P}^{T}\;\right]}^{T}\;\left(P.A\times 1\right)$ and $v_{s,\omega }=q_{sP}⊗g_{\omega }\left(1\times P.A\right)$ and $\sigma_{s}^{2}$ is the receiver noise. Observe that $\varpi_{p}^{T}$ implies that $\left\{a_{pm}\right\}k$ is included in the power spectra composition of PU p; therefore, $\varpi_{s}^{o}$ concatenates the $\left\{a_{pm}\right\}k$ of every PU p. At every time period i, s notices the received power spectra in a discrete frequency $\left\{\omega_{r}\right\}$ in a period $\left[0,\;\pi \right]$ under the size and noise $u_{s,r}$ by mean zero and covariance matrix $C_{u_{s}}$ of size O×O as shown in Equations (3)–(6).

(3) $$b_{s,r}\left(i\right)=v_{s,\omega_{r}}\varpi_{s}^{o}+\sigma_{s}^{2}+u_{s,r},\;\;\;\;\;\;\;\;\;\;\;\;\;r=1,2....O$$

(4) $$b_{s,i}=\left[\begin{array}{c} b_{s,1}\left(i\right)-\sigma_{s}^{2}\\ b_{s,2}\left(i\right)-\sigma_{s}^{2}\\ \vdots \\ \begin{array}{l} b_{s,O-1}\left(i\right)-\sigma_{s}^{2}\\ b_{s,O}\left(i\right)-\sigma_{s}^{2} \end{array} \end{array}\right],\;\;u_{s,i}=\left[\begin{array}{c} u_{s,1}\left(i\right)\\ u_{s,2}\left(i\right)\\ \vdots \\ \begin{array}{l} b_{s,O-1}\left(i\right)\\ b_{s,O}\left(i\right) \end{array} \end{array}\right]$$

(5) $$V_{s,i}=\left[\begin{array}{c} v_{s,\omega_{1}}\\ v_{s,\omega_{2}}\\ \vdots \\ \begin{array}{l} \;\;\;\;\;\;\;\;v_{s,\omega_{o-1}}\\ \;\;\;\;\;\;\;v_{s,\omega_{o}} \end{array} \end{array}\right]=\left[\begin{array}{c} \begin{array}{l} q_{s,i}⊗\;g_{\omega_{1}}\\ q_{s,i}⊗\;g_{\omega_{2}} \end{array}\\ \vdots \\ \begin{array}{l} q_{s,i}⊗\;g_{\omega_{o-1}}\\ q_{s,i}⊗\;g_{\omega_{o}} \end{array} \end{array}\right]$$

(6) $$b_{s,i}=V_{s,i}\varpi_{s}^{o}+u_{s,i}$$

In Equation (6), $\;u_{s}$ refers to model noise and/or measurement with mean zero and $C_{u_{s}}$ of size O×O. At O diverse frequencies, the measurements are taken and therefore, the matrix has O rows. Consequently, in Equation (6), a linear model is attained for computing constraints significance in $\varpi_{s}^{o}$. The steps for processing are described below.

1. The power spectrum of PU, denoted by p, is subjected to path loss attenuation [37].
2. The path loss attenuation is subjected to the total power spectrum and thus, the power spectrum model is obtained.
3. The measurement model per SU s is computed based on the power spectrum of PU, path loss attenuation, and total power spectrum [38], and the model as shown in Equations (7)–(10).
  (7) $$b_{s,r}\left(i\right)=v_{s,\omega_{r}}\varpi_{s}^{o}+\sigma_{s}^{2}+u_{s,r}\left(i\right)\;\;\;\;\;\;\;\;r=1,2......O$$
  (8) $$b_{s,r}\left(i\right)-\sigma_{s}^{2}=v_{s,\omega_{r}}\varpi_{s}^{o}+u_{s,r}\left(i\right)\;\;\;\;\;\;\;\;r=1,2......O$$
  (9) $$b_{s,i}=V_{s,i}\varpi_{s}^{o}+u_{s,i}\;\;\;\;\;\;\;$$

The factor for path loss is modeled as in Equation (10).

(10) $$q_{ps,i}={\left(\frac{b_{ps,i}}{b_{o}}\right)}^{-n}$$

In Equation (10), $b_{ps,i}$ refers to the Euclidean distance from s to p at i; $b_{o}$ refers to a reference distance that is $b_{o}$ = 1; and n designs [39] the attenuation surroundings in CRN [40]. Therefore, the values for path loss among SU s and P PUs are modeled as in Equation (11).

(11) $$q_{s,i}=\left[q_{1s,i},\;q_{2s,i}....q_{Ps,i}\right]$$

In the assessment of $q_{s,i}$, a relevant Gaussian noise of mean zero and SD $\sigma_{q}$ is considered; accordingly, ${\hat{q}}_{s,i}=q_{s,i}+n_{s}$. If SU s changes, $q_{s}$ varies its distance from PUs which also varies accordingly.

For estimating the spectrum, it is adequate to approximate the constraint vector, which factorizes the base operations. Depending upon the network data $\left\{b_{s,i},\;V_{s,i}\right\}$, the issues are treated as an assessment of numerous benefits, and assistance is presumed among the nodes for processing information in a dispersed manner as per the Adapt Then Combine (ATC) policy. The aforesaid policy estimates the centralized outcomes if every node desires to approximate a similar vector of constraints.

Every vector ${\left\{\varpi_{s}^{o}\right\}}_{s=1}^{S}$ includes constraints which are important for the entire model’s constraints of mutual importance to node subset together with other nodes s, and constraints of local importance for node s. In particular, subsets of constraints in $\varpi_{s}^{o}$ account for:

- A global constraint vector associated with the frequency band in power spectra of every PU that impacts every node present in the CRN.
- In a case where J diverse subsets of general constraints is considered, the observation model offered in Equation (6) is rewritten as Equation (12).
  (12) $$b_{s,i}=V_{sf,i}\varpi_{f}^{o}+\sum_{j\in I_{s}}V_{s_{c_{j}},i}\zeta_{s,j}^{o}+u_{s,i}$$

Conventionally, every node tries to resolve by using the subsequent optimization issue [41] as shown in Equation (13).

(13) $$\arg\min\sum_{s=1}^{S}E\left\{{\left\|b_{s,i}-V_{s_{f},i}\varpi_{f}-\sum_{j\in I_{s}}V_{s_{c_{j}},i}\zeta_{s,j}\right\|}^{2}\right\}$$

As per the concept, the amplitude and phase components are considered separately and the optimization issue is defined as shown in Equations (14)–(16) based upon $\varpi_{f}$ and $\zeta_{1}$, $\zeta_{2}$, …, $\zeta_{J}$ in which, $I_{s}$ refers to a well-organized set of index j related with vector $\zeta_{j}$, which is of interest to node s; $V_{s_{f}}$ and $V_{s_{c_{j}}}$ refer to matrices of sizes $O\times M_{f}$ and $O\times M_{c_{j}}$, respectively, and includes columns of $V_{s,i}$ related with $\varpi_{f}$ and $\zeta_{s,j}$.

(14) $$\arg\min\sum_{s=1}^{S}E\left\{\frac{Z_{\alpha \left(d\right)},\;B_{\beta \left(d\right)}}{2}\right\}$$

(15) $$Z_{\alpha \left(d\right)}=1-\tanh\left[\frac{20}{\alpha }\;\log_{10}\left(\frac{\left|X\left(d\right)\right|}{\left|Y\left(d\right)\right|}e^{j\left(b_{s,i}-V_{s_{f},i}\varpi_{f}-\sum_{j\in I_{s}}V_{s_{c_{j}},i}\zeta_{s,j}\right)}\right)\right]$$

(16) $$B_{\beta \left(d\right)}=1-\tanh\left(\frac{b_{s,i}-V_{s_{f},i}\varpi_{f}-\sum_{j\in I_{s}}V_{s_{c_{j}},i}\zeta_{s,j}}{2\pi \alpha }\right)$$

## 4. Component-Specific Adaptive Estimation (CSAE) for MSD Formulation

Here, the diffusion technique ATC which includes an adaptation and a combination phase is exploited. The key phases of the ATC method are as follows:

1. Consider $\phi_{s,\varpi_{f}}^{\left(o\right)},\;{\left\{\phi_{s,\zeta_{j}}^{\left(o\right)}\right\}}_{j\in {Ι}_{s}}$ at every node $s\in \left\{1,2\ldots S\right\}$.
2. For estimating $\varpi_{f}^{o}$ and $\zeta_{j}^{o}$, select O×O combining matrices $R^{\varpi }$ and $R^{\zeta_{j}}$ whose components in every row s are ${\left\{h_{s,l}^{\varpi_{f}}\right\}}_{l=1}^{S}$ and ${\left\{h_{s,l}^{\zeta_{j}}\right\}}_{l=1}^{S}$; fulfill $h_{s,l}^{\varpi_{f}}=0$ if $l\notin \lambda_{s}$ and $\sum_{l\notin \lambda_{s}}h_{s,l}^{\varpi_{f}}=1$; fulfill $h_{s,l}^{\zeta_{j}}=0$, if $l\notin \lambda_{s}\cap \Gamma_{j}$ and $\sum_{l\notin \lambda_{s}\cap \Gamma_{j}}h_{s,l}^{\zeta_{j}}=1$.

The adaptation stage and combination stage at $i^{th}$ iteration is shown in Equations (17) and (18), respectively.

(17) $$\left[\begin{array}{l} \psi_{s}^{\left(i\right)}\\ \zeta_{s}^{\left(i\right)} \end{array}\right]=\left[\begin{array}{l} \varphi_{s,\varpi_{f}}^{\left(i-1\right)}\\ \varphi_{s,\zeta }^{\left(i-1\right)} \end{array}\right]+\mu_{s}V_{s,i}^{H}\left[b_{s,i}-V_{s,i}\left[\begin{array}{l} \varphi_{s,\varpi_{f}}^{\left(i-1\right)}\\ \varphi_{s,\zeta }^{\left(i-1\right)} \end{array}\right]\right]$$

(18) $$\varphi_{s,\varpi_{f}}^{i}=\sum_{l\notin \lambda_{s}}h_{s,l}^{\varpi_{f}}\psi_{l}^{\left(i\right)},\;\varphi_{s,\zeta_{j}}^{\left(i\right)}=\sum_{l\notin \lambda_{s}\cap \Gamma_{j}}h_{s,l}^{\zeta_{j}}\zeta_{l,j}^{\left(i\right)}$$

For every $j\in I_{s}$, $\zeta_{s}^{\left(i\right)}=col\left\{{\left\{\zeta_{s,j}^{\left(i\right)}\right\}}_{j\in I_{s}}\right\}$. When the algorithm ends, $\varphi_{s,\varpi_{f}}^{}$ and $\varphi_{s,\zeta_{j}}^{}k$ approximate the required $\varpi_{f}^{o}$ and $\zeta_{j}^{o}k$. Presuming a clique topology, i.e., $\left|\lambda_{s}\cap \Gamma_{j}\right|=\left|\Gamma_{j}\right|$ for every $s\in \Gamma_{j}$, the even combination rule forms combination weights as in Equations (19) and (20).

(19) $$h_{s,l}^{\varpi_{f}}=\frac{1}{\left|\lambda_{s}\right|}$$

(20) $$h_{s,l}^{\zeta_{j}}=\frac{1}{\left|\lambda_{s}\cap \Gamma_{j}\right|}$$

In conventional work, the adaptive weighting method is deployed as in Equations (21) and (22).

(21) $$\gamma_{s,l}\left(i\right)=\left(1-u\right)\gamma_{s,l}\left(i-1\right)+u{\left\|\psi_{l}^{\left(i\right)}-\varphi_{s,\varpi_{f}}^{\left(i-1\right)}\right\|}^{2}$$

(22) $$\delta_{s,l}\left(i\right)=\left(1-u\right)\delta_{s,l}\left(i-1\right)+u{\left\|\zeta_{l}^{\left(i\right)}-\varphi_{s,\zeta }^{\left(i-1\right)}\right\|}^{2}$$

As per our concept, the amplitude and phase components are considered separately and the adaptive weighting mechanism is defined as shown in Equations (23) and (28).

(23) $$\gamma_{s,l}\left(i\right)=\left(1-u\right)\gamma_{s,l}\left(i-1\right)+u\left(\frac{Z_{\alpha \left(d\right)}-\;B_{\beta \left(d\right)}}{2}\right)$$

(24) $$Z_{\alpha \left(d\right)}=1-\tanh\left[\frac{20}{\alpha }\;\log_{10}\left(\frac{\left|X\left(d\right)\right|}{\left|Y\left(d\right)\right|}e^{j\left(\psi_{l}^{\left(i\right)}-\varphi_{s,\varpi_{f}}^{\left(i-1\right)}\right)}\right)\right]$$

(25) $$B_{\beta \left(d\right)}=1-\tanh\left(\frac{\psi_{l}^{\left(i\right)}-\varphi_{s,\varpi_{f}}^{\left(i-1\right)}}{2\pi \alpha }\right)$$

(26) $$\delta_{s,l}\left(i\right)=\left(1-u\right)\delta_{s,l}\left(i-1\right)+u\left(\frac{Z_{\alpha \left(d\right)}-\;B_{\beta \left(d\right)}}{2}\right)$$

(27) $$Z_{\alpha \left(d\right)}=1-\tanh\left[\frac{20}{\alpha }\;\log_{10}\left(\frac{\left|X\left(d\right)\right|}{\left|Y\left(d\right)\right|}e^{j\left(\zeta_{l}^{\left(i\right)}-\varphi_{s,\zeta }^{\left(i-1\right)}\right)}\right)\right]$$

(28) $$B_{\beta \left(d\right)}=1-\tanh\left(\frac{\zeta_{l}^{\left(i\right)}-\varphi_{s,\zeta }^{\left(i-1\right)}}{2\pi \alpha }\right)$$

(29) $$Z_{\alpha \left(d\right)}=1-\tanh\left[\frac{20}{\alpha }\;\log_{10}\left(\frac{\left|X\left(d\right)\right|}{\left|Y\left(d\right)\right|}e^{j\left(\hat{W}\left(g:r,s,1\right)-W\left(g:r,s,1\right)\right)}\right)\right]$$

(30) $$B_{\beta \left(d\right)}=1-\tanh\left(\frac{\hat{W}\left(g:r,s,1\right)-W\left(g:r,s,1\right)}{2\pi \alpha }\right)$$

(31) $$Z_{\alpha \left(d\right)}=1-\tanh\left[\frac{20}{\alpha }\;\log_{10}\left(\frac{\left|X\left(d\right)\right|}{\left|Y\left(d\right)\right|}e^{j\left(\hat{W}\left(1:m_{t}\left(1\right),l,i-\hat{W}\left(1:m_{t}\left(1\right),s,i-1\right)\right)\right)}\right)\right]$$

(32) $$B_{\beta \left(d\right)}=1-\tanh\left(\frac{\hat{W}\left(1:m_{t}\left(1\right),l,i-\hat{W}\left(1:m_{t}\left(1\right),s,i-1\right)\right)}{2\pi \alpha }\right)$$

(33) $$Z_{\alpha \left(d\right)}=1-\tanh\left[\frac{20}{\alpha }\;\log_{10}\left(\frac{\left|X\left(d\right)\right|}{\left|Y\left(d\right)\right|}e^{j\left(\hat{W}\left(z:y,l,i\;-\hat{W}\left(z:y,s,i-1\;\right)\right)\right)}\right)\right]$$

(34) $$B_{\beta \left(d\right)}=1-\tanh\left(\frac{\hat{W}\left(z:y,l,i\;-\hat{W}\left(z:y,s,i-1\;\right)\right)}{2\pi \alpha }\right)$$

(35) $$Z_{\alpha \left(d\right)}=1-\tanh\left[\frac{20}{\alpha }\;\log_{10}\left(\frac{\left|X\left(d\right)\right|}{\left|Y\left(d\right)\right|}e^{j\left(\hat{W}\left(g:e,s,i\right)-W\left(g:e,s,i\right)\right)}\right)\right]$$

(36) $$B_{\beta \left(d\right)}=1-\tanh\left(\frac{\hat{W}\left(g:e,s,i\right)-W\left(g:e,s,i\right)}{2\pi \alpha }\right)$$

In Equation (23), u refers to a smaller positive value between [0, 1] and $\gamma_{s,l}$ and $\delta_{s,l}$ refers to variance in the evaluation of common and global interest constraints. Subsequently, the weights related to both common and global parameter evaluation process is performed as shown in Equations (37) and (38).

(37) $$h_{s,l}^{\varpi_{f}}\left(i\right)=\frac{\gamma_{s,l}^{-1}\left(i\right)}{\sum_{m\in }\lambda_{s}\gamma_{s,m}^{-1}\left(i\right)}$$

(38) $$h_{s,l}^{\zeta_{j}}\left(i\right)=\frac{\delta_{s,l}^{-1}\left(i\right)}{\sum_{m\in }\lambda_{s}\cap \Gamma_{j}\delta_{s,m}^{-1}\left(i\right)}$$

Algorithms 1 and 2 show the pseudocode for CSAE and MSD estimation.
**Algorithm 1:** Pseudo-code for CSAEOutput: MSD: $S\times \left(J+1\right)\times iter,$$\hat{W}:\;M\times S\times iter$
Input: S, O, M, J, iter, $\mu ,\;B,\;\;\;m_{t},\;\;b,\;\;W,\;\;V_{aug},\;\Gamma$
Step 1: Initialization$\hat{W}=L_{M\times S\times iter},\;\hat{W}\left(:,:,1\right)=randn\left(M,S,1\right)e=L_{O\times S\times iter},MSD=L_{S\times J+1\times iter}$for s=1:S do
 
g=0,  r=0

 for j=1:J+1 do


  g=r+1



  $r=r+m_{t}\left(j\right)$



  $MSD\left(s,j,1\right)=\left(\frac{Z_{\alpha \left(d\right)}-\;B_{\beta \left(d\right)}}{2}\right)$
  
$Z_{\alpha \left(d\right)}$ and $B_{\beta \left(d\right)}$ are computed as shown in Equations (29) and (30)
 endendStep2: Iterative Partfor i=2:iter do
 Adaptation Step for each node
 for s=1:S do

  $e\left(:,s,i\right)=b\left(:,s,i\right)-V_{aug}\left(:,:,s,i\right)\hat{W}\left(:,s,i-1\right)$ do

  
$\hat{W}\left(:,s,i\right)=\hat{W}\left(:,s,i-1\right)+\mu V_{aug}^{H}\left(:,:,s,i\right)e\left(:,s,i\right)$

 end
 for s=1:S do

  Global: Adaptive Weight Estimation

  for l=1:S do


   $\gamma \left(s,l,\;i\right)=\left(1-u\right)\gamma \left(s,l,\;i-1\right)+u\left(\frac{Z_{\alpha \left(d\right)}-\;B_{\beta \left(d\right)}}{2}\right)$
   $Z_{\alpha \left(d\right)}$ and $B_{\beta \left(d\right)}$ are computed as shown in Equation (31) and Equation (32)

  end


  $R^{f}=Rule\left(B,R,\gamma ,3\right)$



  $R^{\varpi }=R^{f}⊗I_{m_{t}\left(1\right)}$


  Elect only global constraint vectors from every user: $\varphi_{\varpi_{f}}=\hat{W}\left(1:m_{t}\left(1\right),:,i\right)$


  Concatenate a global set of constraints from every user $\varphi_{\varpi_{f}}=\varphi_{\varpi_{f}}\left(:\right)$


  Combining step for Global $\varphi_{\varpi_{f}}=R^{\varpi }\varphi_{\varpi_{f}},$



  $\hat{W}\left(1:m_{t}\left(1\right),:,i\right)=reshape\;\left(\varphi_{\varpi_{f}},m_{t}\left(1\right),S\right)$


  General: Adaptive Weights Estimation


  $z=0,\;\;\;y=m_{t}\left(1\right)$


  for j=1:J do


  z=y+1



  $y=y+m_{t}\left(j+1\right)$



   for l=1:S do



    $\delta \left(s,l,\;i\right)=\left(1-u\right)\delta \left(s,l,\;i-1\right)+u\left(\frac{Z_{\alpha \left(d\right)}-\;B_{\beta \left(d\right)}}{2}\right)$
    $Z_{\alpha \left(d\right)}$ and $B_{\beta \left(d\right)}$ are computed as shown in Equations (33) and (34)


   end



   $R^{h_{j}}=Rule\left(B,R,\delta ,4\right)$




   $R^{\zeta_{j}}=R^{h_{j}}⊗I_{m_{t}\left(j+1\right)}$



   Elect userconcerned for $j^{th}$ subset of constraints: $d=find\left(R\left(:,j+1\right)\neq 0\right)$



   Elect $j^{th}$ subset of $M_{h_{j}}$ general constraints from user in $d=\varphi_{\zeta_{j}}=\hat{W}\left(z:y,h,i\right)$



   Concatenate $j^{th}$ subset of general constraints from every user $\varphi_{\zeta_{j}}=\varphi_{\zeta_{j}}\left(:\right)$



   Combining step: $\varphi_{\zeta_{j}}=R^{\zeta_{j}}\varphi_{\zeta_{j}}$




   $\hat{W}\left(z:y,h,i\right)=reshape\left(\varphi_{\zeta_{j}},m_{t}\left(j+1\right),size\left(h,\;\;1\right)\right)$


  end
 endend

**Algorithm 2:** Pseudo code for MSD Estimationfor s=1:S do

 g=0, e=0

 for j=1:J+1 do


  $g=e+1,\;e=e+m_{t}\left(j\right)$


  $MSD\left(s,j,i\right)=\left(\frac{Z_{\alpha \left(d\right)}-\;B_{\beta \left(d\right)}}{2}\right)$
  $Z_{\alpha \left(d\right)}$ and $B_{\beta \left(d\right)}$ are computed as shown in Equations (35) and (36)
 endend

## 5. Results and Discussion

The proposed Component-Specific CSS Model (CSCSSM) was implemented in MATLAB. The CSCSSM was compared to the Priority-Based Two-Stage Detection Model (PBTSDM) [24], Spectrum Binary Particle Swarm Optimization and Queuing Model (SpecBPSO-QM) [28], Optimum Relay Selection and Accurate Cooperative Spectrum Sensing for Hybrid Cognitive Radio Networks (ORS-ACSS) [29], and Adapt-Then-Combine (ATC) method [35]. The CSCSSM and the compared methods were analyzed in terms of Network MSD (dB) by varying the time (*i*). Here, a network with Q = 3 PUs, 5 Pus, and K = 7 SUs, 11 SUs, and 15 SUs was simulated.

### 5.1. Analysis of Network MSD for the CSCSSM and the Conventional Methods with a Network of Q = 3 PUs, and K = 7 SUs, 11 SUs, 15 SUs Simulated while Fixing the σ to 0.05

**Figure 1:**
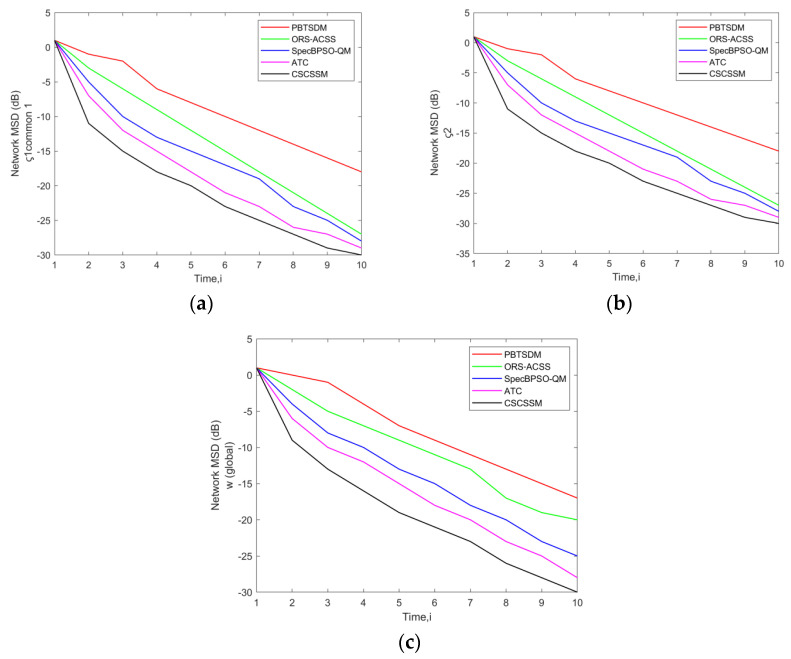
Assessment of network MSD (dB) of the CSCSSM versus traditional schemes for w (global) and Common ς1, ς2 using a network with (**a**) Q = 3 PUs, K = 7 Sus; (**b**) Q = 3 PUs, K = 11 Sus; (**c**) Q = 3 PUs, K = 15 SUs while fixing the σ to 0.05.

In this section, the Network MSD Error evaluation of the CSCSSM was compared to that of the PBTSDM, ORS-ACSS, SpecBPSO-QM, and ATC methods in a network simulated with Q = 3 Pus and K = 7 Sus, 11 Sus, and 15 SUs while adjusting the σ to 0.05 (Figure 1). Also, the time (*i*) was varied from 1 to 10. The MSD error rate must be low for optimal system performance. Time *i* is the time interval. While evaluating Figure 1a, at time 10, the CSCSSM obtained an MSD error of −27.18 dB, whereas the standard methods recorded the highest MSD error rates: PBTSDM with −13.34 dB, ORS-ACSS with −17.84 dB, SpecBPSO-QM with −23.14 dB and ATC with −24.72 dB. In accordance with Figure 1c, the CSCSSM attained an MSD error of −27.91 dB (at time 10), which is extremely lower than PBTSDM (−12.65 dB), ORS-ACSS (−19.74 dB), ATC adaptive weights (−23.46 dB) and ATC (−24.26 dB). The performance of the CSCSSM seems to be more robust than the other standard methods and therefore it attained enhanced performances with a minimal MSD error rate.

### 5.2. Analysis of Network MSD for the CSCSSM and the Conventional Methods with a Network of Q = 3 PUs or 5 PUs and K = 7 SUs, 11 SUs, or 15 SUs Simulated while Fixing the σ to 0.1

**Figure 2:**
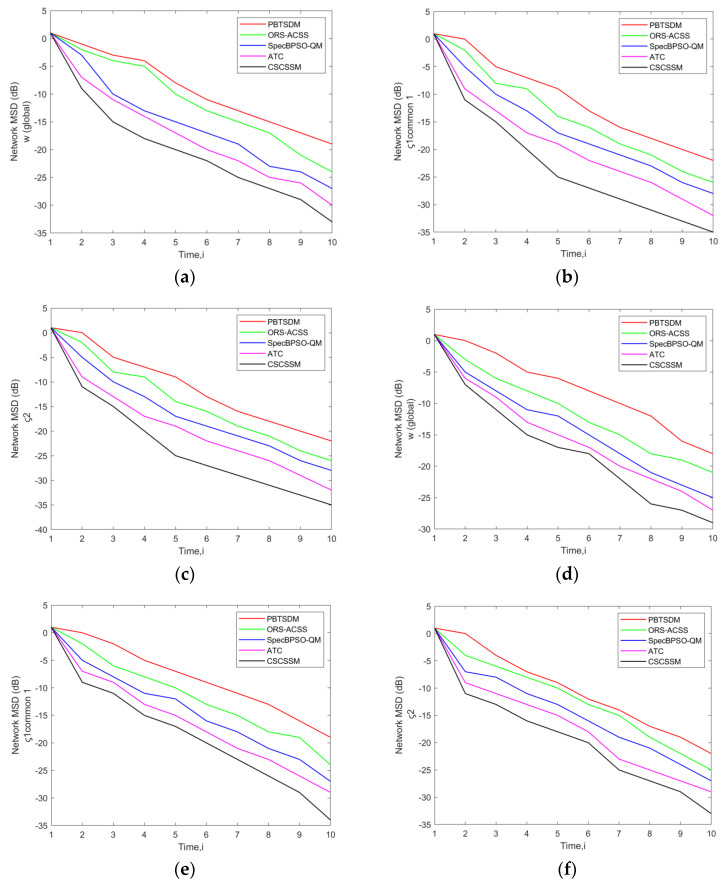
Assessment of network MSD (dB) of the CSCSSM versus traditional schemes for w (global) and Common ς1, ς2 using a network with (**a**) Q = 3 PUs, K = 7 Sus; (**b**) Q = 3 PUs, K = 11 Sus; (**c**) Q = 3 PUs, K = 15 Sus; (**d**) Q = 5 PUs, K = 7 Sus; (**e**) Q = 5 PUs, K = 11 Sus; (**f**) Q = 5 PUs, K = 15 SUs while fixing the σ to 0.1.

The MSD error evaluation of the CSCSSM was compared to that of the PBTSDM, ORS-ACSS, Spec BPSO-QM, and ATC methods by adjusting the σ to 0.1. Also, a network with Q = 3 PUs or 5 PUs, and K = 7 SUs, 11 SUs, or 15 SUs was simulated, and the findings are displayed in Figure 2. On examining Figure 2c, it is evident that the CSCSSM maintained the MSD error value for at the time 9 as approximately −27.48 dB, which is better than PBTSDM with −16.62 dB, ORS-ACSS with −19.78 dB, Spec BPSO-QM with −21.67 dB, and ATC with −23.54 dB. Simultaneously, at time 8, the CSCSSM generated an MSD of −28.42 dB as seen in Figure 2e; meanwhile, the standard methodologies scored the lowest MSD, notably, PBTSDM = −16.84 dB, ORS-ACSS = −18.93 dB, ATC adaptive weights = −19.48 dB, and ATC = −26.74 dB. As a result, the CSCSSM had reduced and minimized MSD errors when compared with the current methodologies.

### 5.3. Analysis of Network MSD for the CSCSSM and the Conventional Methods with a Network of Q = 3 PUs or 5 PUs and K = 7 SUs, 11 SUs, or 15 SUs Simulated While Fixing the σ to 0.2

**Figure 3:**
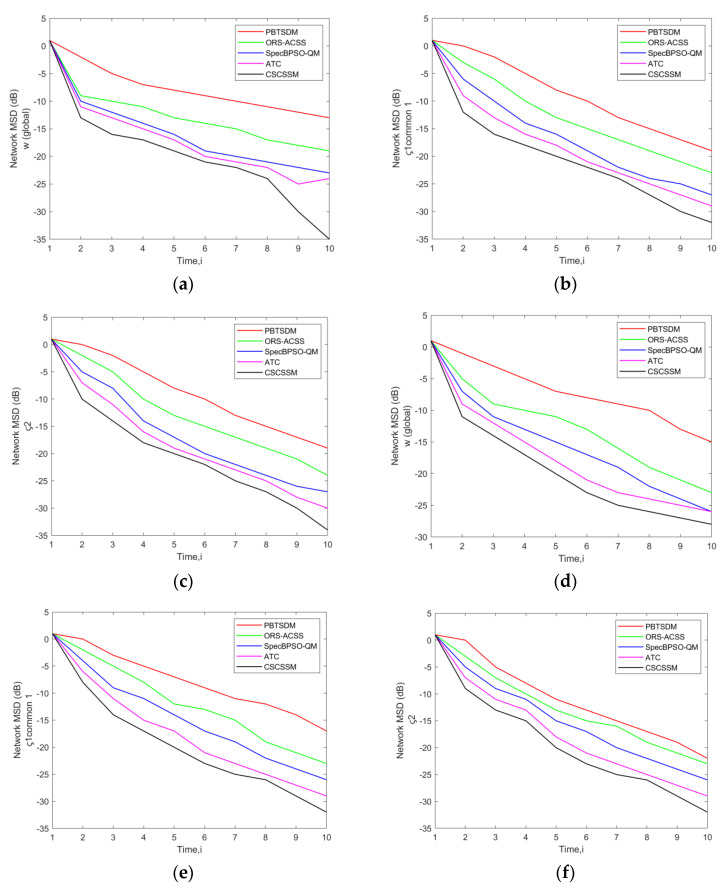
Assessment of network MSD (dB) of the CSCSSM versus traditional schemes for w (global) and Common ς1, ς2 using a network with (**a**) Q = 3 PUs, K = 7 Sus; (**b**) Q = 3 PUs, K = 11 Sus; (**c**) Q = 3 PUs, K = 15 Sus; (**d**) Q = 5 PUs, K = 7 Sus; (**e**) Q = 5 PUs, K = 11 Sus; (**f**) Q = 5 PUs, K = 15 SUs while fixing the σ to 0.2.

The comparison of CSCSSM to PBTSDM, ORS-ACSS, SpecBPSO-QM, and ATC for both datasets is represented in Figure 3. The MSD error evaluation was carried out while fixing the σ to 0.2 and a network was designed to simulate Q = 3 Pus or 5 PUs and K = 7 SUs, 11 SUs, or 15 SUs. According to Figure 3a, the CSCSSM generated an MSD error rate at time 10 of −32.84 dB, while for the PBTSDM, it was −19.56 dB; ORS-ACSS, it was −25.01 dB; SpecBPSO-QM, it was −28.65 dB; and ATC, it was −29.89 dB. Considering Figure 3e at time 7, the models PBTSDM, ORS-ACSS, SpecBPSO-QM, and ATC achieved an MSD error value of −11.24 dB, −19.82 dB, −22.56 dB, and −23.74 dB, although the CSCSSM reported an MSD error of −26.18 dB. This implies the MSD error value is diminished in the CSCSSM in contrast to the previous schemes.

### 5.4. MSD Error Analysis of CSCSSM and Conventional Methods with a Network of Q = 3 PUs or 5 PUs and K = 7 SUs, 11 SUs, or 15 SUs Simulated by Varying the α

The effectiveness of the CSCSSM was assessed compared to the PBTSDM, ORS-ACSS, SpecBPSO-QM, and ATC methods by varying the α from 0.1 to 1 in terms of the MSD error measure and is presented in Table 1. Here, it a network of Q = 3 PUs or 5 PUs and K = 7 SUs, 11 SUs, or 15 SUs was simulated. In particular, while the Q was fixed to 3 PUs and K was fixed as 11 SUs, the CSCSSM recorded an MSD error of −27 dB (α = 0.9), whereas the value for PBTSDM was −13 dB, ORS-ACSS was −21 dB, SpecBPSO-QM was −24 dB, and ATC was −25 dB. For Q = 5 PUs and K = 7 SUs, the CSCSSM had the lowest MSD error rate of −23 dB (α = 0.8). Meanwhile, the conventional methodologies had the highest MSD error values: PBTSDM (0.8 dB), ORS-ACSS (−13 dB), SpecBPSO-QM (−17 dB), and ATC (−20 dB). The CSCSSM performed well in the MSD error measurements compared to the conventional algorithms, indicating that the MSD errors of the established algorithms is extremely high.

The proposed component−specific cooperative spectrum sensing model (CSCSSM) outperformed the existing methods because the proposed model handles the interrupted secondary users’ requirement to switch operating channels. With this CSCSSM model, the interactions between several channels are precisely described. Additionally, this model uses the simultaneous consideration of traffic patterns and target channel selection strategies on transmission latency to avoid spectrum handoff in CRNs. The collected results demonstrate that the performance of the negotiated and opportunistic spectrum access strategies vary noticeably. The proposed CSCSSM and the outcomes are very beneficial for CRN optimization.

## 6. Conclusions

This paper implemented a new CSCSSM in CRNs. In the past, it was customary to calculate the error between direct signal vectors. If phase shift or amplitude minimization takes place, the error will be large and information will be lost. In order to reduce information loss, a component−specific (amplitude and phase component) system model for signal estimation was formulated. At time 10, the MSD error rate produced by the CSCSSM was −32.84 dB, compared to −19.56 dB for the PBTSDM, −25.01 dB for the ORSACSS, −28.65 dB for the SpecBPSO−QM, and −29.89 dB for the ATC. The models PBTSDM, ORSACSS, SpecBPSO−QM, and ATC obtained MSD error values of −11.24 dB, −19.82 dB, −22.56 dB, and −23.74 dB, respectively, while the CSCSSM recorded an MSD error of −26.18 dB at time 7. This implies that the MSD error value was diminished in the CSCSSM when compared to the previous schemes.

## Figures and Tables

**Table 1:** MSD error analysis of CSCSSM versus traditional methods with a network of Q = 3 PUS, 5 PUs and K = 7 SUs, 11 SUs, 15 Sus, simulated by varying the α.

**Q = 3 PUs and K = 7 SUs**					
**α**	**PBTSDM**	**ORS-ACSS**	**ATC Adaptive Weights**	**ATC**	**CSCSSM**
0.1	−5	−7	−8	−9	−10
0.2	−6	−9	−10	−11	−13
0.3	−7	−10	−12	−13	−16
0.4	−8	−11	−14	−15	−17
0.5	−9	−13	−16	−17	−19
0.6	−10	−14	−19	−20	−21
0.7	−11	−15	−20	−21	−22
0.8	−12	−17	−21	−22	−24
0.9	−13	−18	−22	−23	−25
1	−14	−19	−23	−24	−27
**Q = 3 PUs and K = 11 SUs**					
**α**	**PBTSDM**	**ORS-ACSS**	**ATC Adaptive Weights**	**ATC**	**CSCSSM**
0.1	−4	−6	−7	−8	−10
0.2	−5	−8	−10	−9	−11
0.3	−6	−9	−11	−12	−14
0.4	−7	−10	−13	−15	−17
0.5	−8	−11	−15	−18	−20
0.6	−9	−13	−17	−21	−23
0.7	−10	−16	−19	−23	−25
0.8	−11	−19	−22	−24	−26
0.9	−13	−21	−24	−25	−27
1	−15	−23	−26	−26	−28
**Q = 3 PUs and K = 15 SUs**					
**α**	**PBTSDM**	**ORS-ACSS**	**ATC Adaptive Weights**	**ATC**	**CSCSSM**
0.1	−4	−5	−7	−8	−9
0.2	−6	−7	−8	−9	−13
0.3	−7	−8	−10	−12	−15
0.4	−8	−9	−13	−15	−18
0.5	−9	−12	−15	−18	−20
0.6	−10	−15	−17	−21	−23
0.7	−12	−18	−19	−23	−25
0.8	−14	−21	−23	−26	−27
0.9	−16	−24	−25	−27	−29
1	−18	−27	−28	−29	−30
**Q = 5 PUs and K = 7 SUs**					
**α**	**PBTSDM**	**ORS-ACSS**	**ATC Adaptive Weights**	**ATC**	**CSCSSM**
0.1	0.1	−5	−6	−8	−10
0.2	0.2	−6	−7	−11	−12
0.3	0.3	−7	−8	−12	−13
0.4	0.4	−8	−9	−13	−14
0.5	0.5	−9	−10	−14	−15
0.6	0.6	−10	−11	−15	−18
0.7	0.7	−11	−13	−18	−20
0.8	0.8	−13	−17	−20	−23
0.9	0.9	−15	−19	−23	−25
1	1	−17	−20	−25	−28
**Q = 5 PUs and K = 11 SUs**					
**α**	**PBTSDM**	**ORS-ACSS**	**ATC Adaptive Weights**	**ATC**	**CSCSSM**
0.1	−6	−7	−8	−9	−10
0.2	−7	−8	−9	−10	−12
0.3	−8	−9	−10	−11	−13
0.4	−9	−10	−13	−14	−17
0.5	−10	−11	−15	−17	−19
0.6	−11	−13	−17	−20	−21
0.7	−13	−15	−19	−22	−24
0.8	−15	−17	−23	−25	−26
0.9	−17	−20	−25	−27	−28
1	−19	−25	−28	−29	−31
**Q = 5 PUs and K = 15 SUs**					
**α**	**PBTSDM**	**ORS-ACSS**	**ATC Adaptive Weights**	**ATC**	**CSCSSM**
0.1	−4	−5	−6	−7	−8
0.2	−6	−7	−8	−9	−10
0.3	−7	−8	−9	−11	−13
0.4	−8	−9	−10	−13	−15
0.5	−9	−10	−13	−15	−17
0.6	−10	−13	−15	−17	−19
0.7	−11	−15	−18	−20	−23
0.8	−12	−18	−21	−22	−26
0.9	−16	−19	−23	−24	−27
1	−18	−21	−25	−27	28

## Data Availability

Not applicable.

## Abbreviations

**Abbreviation**	**Description**
ACSS	Attack Aware CSS
ANN	Artificial Neural Network
ATC	Adapt Then Combine
CBCSS	Cluster−Based CSS
CH	Cluster Head
CR	Cognitive Radio
CRN	Cognitive Radio Networks
CSAE	Component−Specific Adaptive Estimation
CSCSSM	Component−Specific CSS Model
CSMA	Carrier Sense Multiple Access
CSS	Cooperative Spectrum Sensing
CSAE	Component−Specific Adaptive Estimation
CSMA	Carrier Sense Multiple Access
ED	Energy Detector
EE	Energy Efficiency
FA	False Alarm
HD	Hybrid Detector
MAC	Media Access Control
MD	Matched Detector
MRC	Multiple Reporting Channel
ORS	Optimal Relay Selection
PBTSDM	Priority−Based Two−Stage Detection Model
PU	Primary User
QoS	Quality of Service
RACRN	Random Access CRN
RF	Radio Frequency
SD	Standard Deviation
SE	Spectral Efficiency
Spec BPSO	Spectrum Binary Particle Swarm Optimization
SNR	Signal−to−Noise Ratio
SS	Spectrum Sensing
SU	Secondary User
WRAN	Wireless Regional Area Network

## References

[1] Nkalango S.D.A., Zhao H., Song Y., Zhang T. (2020). Energy efficiency under double deck relay assistance on cluster cooperative spectrum sensing in hybrid spectrum sharing. IEEE Access.

[2] Goudos S.K., Siakavara K., Sahalos J.N. (2014). Novel spiral antenna design using artificial bee colony optimization for UHF RFID Applications. IEEE Antennas Wirel. Propag. Lett..

[3] Eappen G., Shankar T., Nilavalan R. (2022). Cooperative relay spectrum sensing for cognitive radio network: Mutated MWOA-SNN approach. Appl. Soft. Comput..

[4] Kim J., Choi J.P. (2019). Sensing coverage-based cooperative spectrum detection in cognitive radio networks. IEEE Sens. J..

[5] Goudos S.K., Tsiflikiotis A., Babas D., Siakavara K., Kalialakis C., Karagiannidis G.K. Evolutionary Design of a Dual Band E-Shaped Patch ANTENNA for 5G Mobile Communications. Proceedings of the 6th International Conference on Modern Circuits and Systems Technologies, MOCAST 2017.

[6] Nasser A., Hassan H.A.H., Chaaya J.A., Mansour A., Yao K.-C. (2021). Spectrum Sensing for Cognitive Radio: Recent Advances and Future Challenge. Sensors.

[7] Patel A., Ram H., Jagannatham A.K., Varshney P.K. (2018). Robust cooperative spectrum sensing for MIMO cognitive radio networks under CSI uncertainty. IEEE Trans. Signal Process..

[8] Goudos S.K., Tsoulos G.V., Athanasiadou G., Batistatos M.C., Zarbouti D., Psannis K.E. (2019). Artificial neural network optimal modeling and optimization of UAV measurements for mobile communications using the L-SHADE algorithm. IEEE Trans. Antennas Propag..

[9] Feng J., Li S., Lv S., Wang H., Fu A. (2018). Securing cooperative spectrum sensing against collusive false feedback attack in cognitive radio networks. IEEE Trans. Veh. Technol..

[10] Bagheri A., Ebrahimzadeh A. (2021). Statistical analysis of lifetime in wireless cognitive sensor network for multi-channel cooperative spectrum sensing. IEEE Sens. J..

[11] Gao A., Du C., Ng S.X., Liang W. (2021). A cooperative spectrum sensing with multi-agent reinforcement learning approach in cognitive radio networks. IEEE Commun. Lett..

[12] Wu W., Wang Z., Yuan L., Zhou F., Lang F., Wang B., Wu Q. (2021). IRS-enhanced energy detection for spectrum sensing in cognitive radio networks. IEEE Wirel. Commun. Lett..

[13] Agrawal S.K., Samant A., Yadav S.K. (2022). Spectrum sensing in cognitive radio networks and metacognition for dynamic spectrum sharing between radar and communication system: A review. Phys. Commun..

[14] Goudos S.K., Diamantoulakis P.D., Karagiannidis G.K. (2018). Multi-objective optimization in 5G wireless networks with massive MIMO. IEEE Commun. Lett..

[15] Kumar A., Pandit S., Singh G. (2021). Threshold selection analysis of spectrum sensing for cognitive radio network with censoring based imperfect reporting channels. Wirel. Netw..

[16] Jin Z., Yao K., Lee B., Cho J., Zhang L. (2019). Channel status learning for cooperative spectrum sensing in energy-restricted cognitive radio networks. IEEE Access.

[17] Alhamad R., Wang H., Yao Y.-D. (2017). Cooperative spectrum sensing with random access reporting channels in cognitive radio networks. IEEE Trans. Veh. Technol..

[18] Reddy S.S., Prasad M.S.G. (2020). Improved Whale Optimization Algorithm and Convolutional neural network based Cooperative Spectrum Sensing in Cognitive Radio Networks. Inf. Secur. J. Glob. Perspect..

[19] Olawole A.A., Takawira F., Oyerinde O.O. (2019). Cooperative spectrum sensing in multichannel cognitive radio networks with energy harvesting. IEEE Access.

[20] Shen F., Ding G., Wang Z., Wu Q. (2019). UAV-Based 3D Spectrum Sensing in Spectrum-Heterogeneous Networks. IEEE Trans. Veh. Technol..

[21] Thareja Y., Sharma K.K. (2021). A posterior transition probability-based model for spectrum sensing in cognitive radio networks for maximized network lifetime and performance enhancement. Int. J. Commun. Syst..

[22] Chatterjee S., Maity S.P., Acharya T. (2019). Energy-spectrum efficiency trade-off in energy harvesting cooperative cognitive radio networks. IEEE Trans. Cognit. Commun. Netw..

[23] Akyildiz I.F., Lo B.F., Balakrishnan R. (2011). Cooperative spectrum sensing in cognitive radio networks: A survey. Phys. Commun..

[24] Muthukkumar R., Manimegalai D. (2018). Enhancing cooperative spectrum sensing in cognitive radio ad hoc networks using priority-based two-stage detection model. Wirel. Netw..

[25] Atmaca S., Şayli Ö., Yuan J., Kavak A. (2017). Throughput maximization of CSMA in cognitive radio networks with cooperative spectrum sensing. Wirel. Pers. Commun..

[26] Sharifi A.A. (2019). Attack-aware defense strategy: A robust cooperative spectrum sensing in cognitive radio sensor networks. Iran. J. Sci. Technol. Trans. Electr. Eng..

[27] Ye H., Jiang J. (2021). Optimal linear weighted cooperative spectrum sensing for clustered-based cognitive radio networks. EURASIP J. Wirel. Commun. Netw..

[28] Devi M.K., Umamaheswari K. (2021). Optimization techniques for spectrum handoff in cognitive radio networks using cluster based cooperative spectrum sensing. Wirel. Netw..

[29] Rajaganapathi R., Nathan P.M. (2020). ORS-ACSS: Optimum relay selection and accurate cooperative spectrum sensing for hybrid cognitive radio networks. Wirel. Pers. Commun..

[30] Hossain M.A., Schukat M., Barrett E. (2021). Enhancing the spectrum sensing performance of cluster-based cooperative cognitive radio networks via sequential multiple reporting channels. Wirel. Pers. Commun..

[31] Jaglan R.R., Mustafa R., Agrawal S. (2018). Scalable and robust ANN based cooperative spectrum sensing for cognitive radio networks. Wirel. Pers. Commun..

[32] Arshid K., Jianbiao Z., Hussain I., Pathan M.S., Yaqub M., Jawad A., Munir R., Ahmad F. (2022). Energy efficiency in cognitive radio network using cooperative spectrum sensing based on hybrid spectrum handoff. Egypt Inf. J..

[33] Bani K., Kulkarni V. (2022). Hybrid spectrum sensing using MD and ED for cognitive radio networks. J. Sens. Actuat. Netw..

[34] Plata-Chaves J., Bogdanović N., Berberidis K. (2015). Distributed diffusion-based LMS for node-specific adaptive parameter estimation. IEEE Trans. Signal Process..

[35] Trigka M., Dritsas E. (2022). An efficient distributed approach for cooperative spectrum sensing in varying interests cognitive radio networks. Sensors.

[36] Boulogeorgos A.A.A., Salameh H.A.B., Karagiannidis G.K. (2017). Spectrum sensing in full-duplex cognitive radio networks under hardware imperfections. IEEE Trans. Veh. Technol..

[37] Chavarro-Barrera L., Perez-Londono S., Mora-Florez J. (2021). An Adaptive Approach for Dynamic Load Modeling in Microgrids. IEEE Trans. Smart Grid.

[38] Zaeemzadeh A., Joneidi M., Rahnavard N., Qi G.-J. (2018). Co-SpOT: Cooperative spectrum opportunity detection using Bayesian clustering in spectrum-heterogeneous cognitive radio networks. IEEE Trans. Cognit. Commun. Netw..

[39] Saifan R., Jafar I., Al Sukkar G. (2017). Optimized cooperative spectrum sensing algorithms in cognitive radio networks. Comput. J..

[40] Liu X., Zheng K., Chi K., Zhu Y.-H. (2020). Cooperative Spectrum Sensing Optimization in Energy-Harvesting Cognitive Radio Networks. IEEE Trans. Wirel. Commun..

[41] Goudos S.K. (2007). A versatile software tool for microwave planar radar absorbing materials design using global optimization algorithms. Mater. Des..

